# Functional Outcomes After Buddy Tape Immobilization in a Healthy Population

**DOI:** 10.1016/j.jhsg.2026.100960

**Published:** 2026-02-24

**Authors:** Peter Joo, Jonathan Cui, Max Modrak, Neil Pathak, Giscard Adeclat, Rey Ramirez

**Affiliations:** Department of Orthopaedics & Rehabilitation, Yale School of Medicine, New Haven, CT

**Keywords:** Buddy tape, Finger injury, Finger trauma, Metacarpal fracture, Phalangeal fracture, Typing

## Abstract

**Purpose:**

Buddy taping is a simple, cheap, and common method of treatment for various hand conditions. Unlike static splinting, buddy taping allows for maintained range of motion during the period of treatment. This study aims to objectively measure the functional impact that buddy tape immobilization has on activities of daily living.

**Methods:**

Healthy volunteers completed a baseline typing assessment and Patient Reported Outcome Measurement Information System Upper Extremity (PROMIS-UE) functional assessment. The participants then repeated the typing assessment and PROMIS-UE assessment while two of their fingers were buddy taped in all possible permutations (index to middle, middle to ring, ring to small) in both the dominant and nondominant hands. Volunteers acted as their own control group. Differences in overall average typing speed (words per minute [WPM]), accuracy, and PROMIS-UE scores were evaluated via one-way analysis of variance or two-sample *t* tests.

**Results:**

Twenty-seven healthy volunteers were recruited. There were statistically significant decreases in typing speed with all buddy tape configurations, ranging from 75.4 WPM to 44.8 WPM as well as accuracy ranging from 92.6% to 84.8%. The most affected taping configuration was buddy tape between the left middle and index fingers, and the least affected taping configuration was buddy tape between the right-small and -ring fingers. There were no statistically significant differences in PROMIS-UE scores for any buddy taping configuration.

**Conclusions:**

This study demonstrates that buddy taping alone significantly decreases typing speed and accuracy, particularly when the left index and middle fingers are immobilized, though overall self-reported function remains unaffected. These findings provide objective, quantifiable measurements of disability and can help provide clear expectations to patients, employers, or insurers regarding the temporary limitations imposed by buddy taping.

**Level of evidence:**

III, nonconsecutive cohort study.

Buddy taping is commonly employed as a simple, cost-effective, and safe method of relative finger splinting that allows for controlled early range of motion.^1^^,^^2^ It is used for many conditions in hand surgery, such as metacarpal fractures, for which is has shown superior outcomes to alternatives such as cast immobilization.^3^

Computers and/or keyboards are needed for many jobs. The need to be able to use a keyboard has increased in recent years.^4^^,^^5^ Patients being treated for hand conditions often seek information on how limited they will be at work. However, there is limited quantitative guidance for patients being treated with buddy taping on how it will affect their ability to type and perform at their jobs.

This study aims to investigate the functional impact that buddy tape immobilization has on activities of daily living, and more specifically on use of a keyboard. We hypothesize that buddy taping will decrease the ability to use a keyboard. This will be demonstrated by decreased performance on tests of typing speed and accuracy. We hypothesize that buddy taping will decrease global use of the hand. This will be demonstrated by decreased functional outcome measures for use of the upper extremity.

## Materials and Methods

This study was deemed low risk and Institutional Review Board approval was obtained. All participants signed informed consent prior to participation in the study. No Health Insurance Portability and Accountability Act consent was needed as no identifying medical information was collected.

Healthy volunteers without any history of functional limitations of the hands or fingers were recruited. Exclusion criteria were any minors under the age of 18 years, those with known functional hand deficits or limitations, and those unable to perform multiple typing assessments or fill out the required patient-reported outcomes assessments. Demographic data were collected for all participants, including age, sex, hand dominance, typing frequency, and typing style (touch type using all 10 fingers, hybrid, or hunt-and-peck typing using one or two fingers).

These healthy volunteers completed a baseline 1-minute typing assessment (monkeytype.com), as well as a baseline Patient Reported Outcome Measurement Information System Upper Extremity (PROMIS-UE) functional assessment. The participants then repeated the typing and PROMIS-UE assessments while two of their fingers were buddy taped in the following permutations: left hand index to middle finger (L23), left hand middle to ring finger (L34), left hand ring to small finger (L45), right-hand index to middle finger (R23), right-hand middle to ring finger (R34), and right-hand ring to small finger (R45). The 1-minute typing assessments were randomized during each repeated test to prevent sequential learning and improvement, and limit bias. The typing speed was measured in words per minute (WPM), and the typing accuracy was measured and reported by the assessment tool. The relative percent change was calculated using the formula 1-(group/control) = % change.

Each participant acted as their own control group. Differences in overall average typing speed, accuracy, and PROMIS-UE scores were evaluated via one-way analysis of variance (ANOVA) and differences in the same three parameters between the control and individual taping configurations were evaluated pairwise by *t* tests. Alpha level was set at the conventional 0.05, and beta level at 0.20, with *P* < .05 representing statistical significance. An a priori power analysis was performed given an average typing speed of 40 WPM and a minimum 10 WPM between-group difference in speed and SD of eight indicated that a minimum sample size of 11 subjects per group was needed to yield the statistical power per above.

## Results

Overall, 27 healthy volunteers were recruited for the study. The demographic data are summarized in the Table. Of note, while all participants noted daily keyboard use, only 54% used the Touch Type method.

**Table.** Demographic Data

Demographics		
Variable	N/Value	%
Total	27	100
Age, y (average, range)	32.12	(26−61)
M	15	55.6
Right-hand dominant	26	96.3
Daily keyboard use	27	100.0
Typing method		
Touch type	14	51.9
Hybrid	9	33.3
Hunt-and-peck	4	14.8

**Figure 1:**
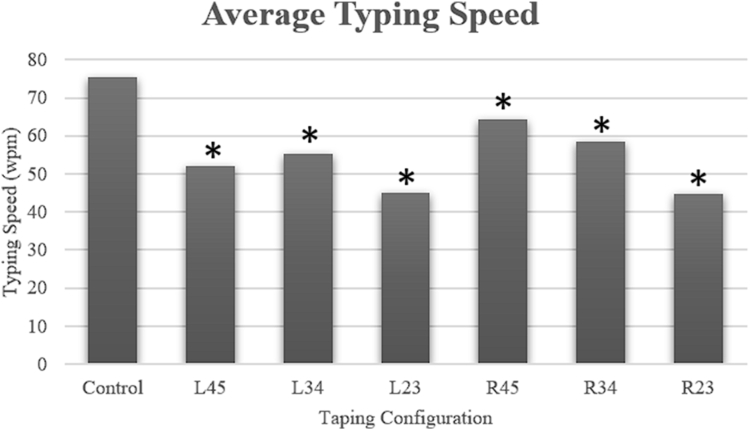
Average typing speed in WPM across the different buddy taping configurations. ∗indicates statistical significance compared to the control (*P* < .05).

There was an overall statistically significant difference in average typing speeds between the various buddy tape configurations (Fig. 1). Without any restrictions, the average typing speed was 75.4 WPM. In descending order, the R45 (64.4 WPM, 14.5% slower), R34 (58.5 WPM, 22.3% slower), L34 (55.4 WPM, 26.5% slower), L45 (52.0 WPM, 31.1% slower), R23 (44.9 WPM, 40.4% slower), and L23 (44.8 WPM, 40.6% slower) buddy taping configurations were noted to be significantly different on ANOVA (*P* < .001).

**Figure 2:**
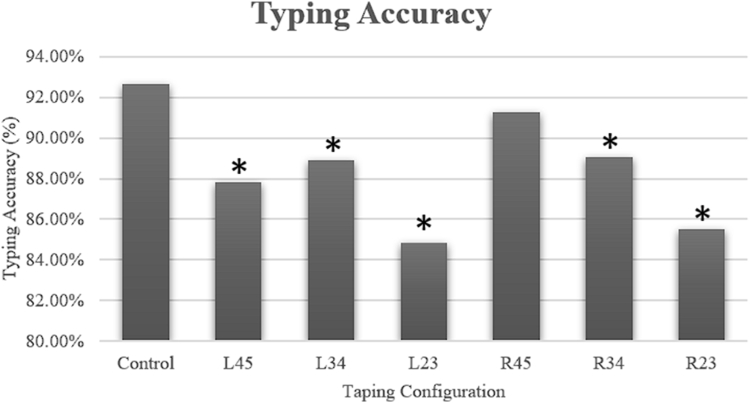
Typing accuracy across different buddy taping configurations. ∗indicates statistical significance compared to the control (*P* < .05).

There was an overall statistically significant difference in average typing accuracy between the various buddy tape configurations (Fig. 2). Without any restrictions, the average typing accuracy was 92.6%. In descending order, the R45 (91.3%), R34 (89.1%), L34 (88.9%), L45 (87.8%), R23 (85.5%), and L23 (84.8%) buddy taping configurations were noted to be significantly different on ANOVA (*P* < .001).

We note that the buddy tape configuration that affected typing speed the most (L23) also affected accuracy the most, and the buddy tape configuration that affected typing speed the least (R45) affected accuracy the least. In other words, the order of buddy tape configurations from most impactful to least impactful was the same for typing speed as it was for accuracy.

**Figure 3:**
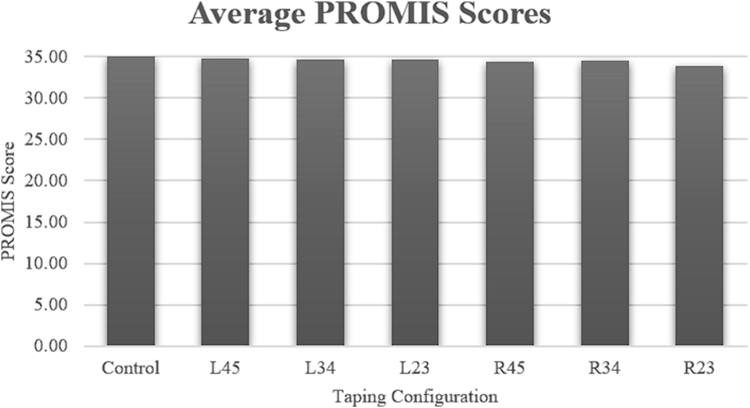
Average Patient Reported Outcome Measurement Information System (PROMIS) scores across the different buddy taping configurations.

Regarding the PROMIS-UE assessments, there were no statistically significant differences between the control and experimental groups (Fig. 3). Without any restrictions, the average PROMIS-UE score was 35. In descending order, the L45 (34.7), L34 (34.6), L23 (34.6), R34 (34.5), R45 (34.4), and R23 (33.9) buddy taping configurations were not noted to be statistically significant on ANOVA (*P* = .713).

## Discussion

This study quantitatively assesses the functional outcomes of buddy tape immobilization on typing speed and accuracy in a healthy population. While buddy taping is widely used in the management of various conditions, few studies have examined its effect on fine motor tasks required in modern occupational settings.^6^^,^^7^ The growing reliance on computer-based work underscores the importance of understanding how common immobilization strategies may impact activities such as typing, a daily activity for much of the workforce.^5^ By focusing on this specific domain, our findings provide practical insights that can directly inform patient counseling and expectations following finger injuries.

Our results demonstrate buddy taping significantly reduced both typing speed and accuracy, with the greatest impairment observed when the left index and middle fingers were taped together. This finding aligns with prior research on hand biomechanics, which highlights the critical role of the index and middle fingers in typing efficiency and dexterity.^8^^,^^9^ While most subjects were right handed, hand dominance did not appear to affect results in this study, perhaps due to the fact that most keyboard configurations do not take into account the right handedness versus left handedness of the user.

Previous studies have reported that splinting or immobilization of the fingers can lead to short-term decreases in fine motor function, though long-term functional outcomes are often preserved with early mobilization strategies, such as buddy taping.^6^^,^^10^^,^^11^ Our study extends this knowledge by providing objective, quantitative evidence that buddy taping does hinder typing performance.

Our study did not find that buddy taping significantly affected patient-reported upper extremity function (PROMIS-UE scores). This suggests that buddy taping alone does not cause major functional deficits in daily activities. However, an alternative conclusion is that our data show that the PROMIS-UE is not a good test for assessing ability to use a keyboard. It is notable that none of the questions on the PROMIS-UE 2.0 specifically ask about keyboard or computer usage.^12^ Thus, metrics such as “words per minute” may be better assessments for keyboard use than PROMIS.

Limitations must be considered. The study population consisted of healthy volunteers rather than patients recovering from fractures, which may limit generalizability to injured populations. Clearly, patients with significant hand injuries will face additional barriers to keyboard usage than just the buddy tape. It may be that, when looking at a specific injury (eg, metacarpal fracture), patients with buddy tape perform worse than, equal to, or even better than patients without buddy tape. Additionally, the short-term cross-sectional nature of the assessments may not reflect long-term adaptation to wearing buddy tape, as users might compensate for the restriction over time. However, this is probably a fair representation, as most people are likely to be asked to wear buddy tape without forewarning and for only a short period. Finally, the typing test employed provides only a limited snapshot of hand function and may not fully capture the broader impact of buddy taping on occupational performance or daily tasks.

The strengths of our study are that the within-subject design minimized interindividual variability and improved the reliability of observed differences across taping configurations. Randomization of typing passages also reduced learning bias.

In conclusion, this study demonstrates that buddy taping alone significantly decreases typing speed and accuracy, particularly when the left index and middle fingers are immobilized, though overall self-reported functional outcome measures remain unaffected. These findings highlight the importance of providing patients, especially those in occupations reliant on typing, with clear expectations regarding the temporary limitations imposed by buddy taping.

## Conflicts of Interest

No benefits in any form have been received or will be received related directly to this article.
